# State Tobacco Control Program Spending — United States, 2011

**Published:** 2015-06-26

**Authors:** Jidong Huang, Kimp Walton, Robert B. Gerzoff, Brian A. King, Frank J. Chaloupka

**Affiliations:** 1 Health Policy Center, University of Illinois at Chicago, Illinois; 2 Office on Smoking and Health, National Center for Chronic Disease Prevention and Health Promotion, CDC; 3 Department of Economics and School of Public Health, University of Illinois at Chicago, Illinois

Evidence-based, statewide tobacco control programs that are comprehensive, sustained, and accountable reduce smoking rates and tobacco-related diseases and deaths (1,2). States that made larger investments in tobacco prevention and control have seen larger declines in cigarettes sales than the United States as a whole (3), and the prevalence of smoking has declined faster as spending for tobacco control programs has increased (4,5). CDC’s *Best Practices for Comprehensive Tobacco Control Programs* (Best Practices) outlines the elements of an evidence-based state tobacco control program and provides recommended state funding levels to substantially reduce tobacco-related disease, disability, and death (1,2). To analyze states’ spending in relation to program components outlined within Best Practices, CDC assessed state tobacco control programs’ expenditures for fiscal year 2011. In 2011, states spent approximately $658 million on tobacco control and prevention, which accounts for less than 3% of the states’ revenues from the sale of tobacco products and only 17.8% of the level recommended by CDC.* Evidence suggests that funding tobacco prevention and control efforts at the levels recommended in Best Practices could achieve larger and more rapid reductions in tobacco use and associated morbidity and mortality (2,3).

Following CDC’s first publication of Best Practices in 1999, overall funding for state tobacco control programs has more than doubled, and states restructured their tobacco control programs to align with CDC’s goals and programmatic recommendations (2). The 1999 report recommended that states invest a combined $1.6 to $4.2 billion annually in such programs. In 2007, the recommendation was updated to $3.7 billion annually (1). These recommendations were updated again in 2014 ($3.3 billion) to reflect additional state experiences in implementing comprehensive tobacco control programs, new scientific literature, and changes in state populations, inflation, media costs, Affordable Care Act effects, and the national tobacco control landscape (2). To date, all 50 states and the District of Columbia (DC) have state tobacco control programs that are funded through various revenue streams, including tobacco industry master settlement payments to states, cigarette excise tax revenues, state general funds, federal government funds, and nonprofit organizations.†

For this analysis, researchers from the Health Policy Center at the University of Illinois at Chicago obtained reports of state tobacco control programs’ expenditures for fiscal year 2011 for all 50 states and DC. They directly contacted representatives within relevant state organizations and agencies, and accessed their websites.§ When multiple agencies and organizations were responsible for a state’s tobacco control program, expenditures from each organization were combined. In addition to total tobacco control expenditures, expenditure data were collected for the five program components outlined in Best Practices (2007): 1) state and community interventions; 2) health communication interventions; 3) cessation interventions; 4) surveillance and evaluation; and 5) administration and management (1).¶ Expenditures for the United States and for each state were calculated by program component, as overall, per capita, and percentage of recommended funding levels in Best Practices (2007).**

In fiscal year 2011, combined expenditures by all 50 states and DC for tobacco prevention and control activities totaled $658.15 million (Table 1); by state, overall expenditures ranged from $1.68 million in New Hampshire to $94.66 million in California. By program component, combined expenditures by all 50 states and DC were $272.38 million for state and community interventions, $123.53 million for health communication interventions, $134.09 million for cessation interventions, $61.35 million for surveillance and evaluation, and $66.79 million for administration and management.

Combined expenditures by all 50 states and DC for tobacco prevention and control activities were $2.11 per capita (Table 2); by state, per capita expenditures ranged from $0.33 in Tennessee to $14.74 in Alaska. By program component, combined per capita expenditures by all 50 states and DC were $0.87 for state and community interventions, $0.40 for health communication interventions, $0.43 for cessation interventions, $0.20 for surveillance and evaluation, and $0.21 for administration and management.

Combined expenditures by all 50 states and DC for tobacco prevention and control activities were 17.8% of the level recommended by CDC (Table 3). Eight states spent 50% or more of the recommended level (Alaska, Colorado, Delaware, Hawaii, Montana, North Dakota, Oklahoma, and Wyoming), while 13 states (Connecticut, Georgia, Kansas, Kentucky, Maryland, Massachusetts, Michigan, New Hampshire, New Jersey, Ohio, South Carolina, Tennessee, and Texas) spent less than 10% of the recommended level. By program component, expenditures as a percentage of the recommended amount were 18.6% for state and community interventions, 17.5% for health communication interventions, 12.8% for cessation interventions, 19.1% for surveillance and evaluation, and 41.5% for administration and management.

## Discussion

The findings in this report reveal that state investments in tobacco prevention and control programs in fiscal year 2011 were considerably less than levels recommended in CDC’s Best Practices (1,2). In 2011, states spent only $658 million (<3% of $24.2 billion they received from tobacco tax revenues and Master Settlement Agreement payments^*^) in tobacco control and prevention activities, compared with the $8.8 billion that tobacco companies spent on cigarette and smokeless tobacco advertising and promotion that year (6,7). Despite significant declines in cigarette smoking in recent years, 17.8% of U.S. adults and 15.7% of high school students still smoke cigarettes (8,9). Moreover, the prevalence of use of other tobacco products such as cigars and smokeless tobacco has not changed (3), and the prevalence of use of emerging products, including electronic cigarettes (e-cigarettes) and hookah, has rapidly increased (3). Investing in comprehensive tobacco control programs and implementing evidence-based interventions have been shown to reduce youth initiation, tobacco-related disease and death, and tobacco-related health care costs and lost productivity. Moreover, if states allocate funding for tobacco control at CDC’s Best Practices levels, they have the potential to achieve larger and more sustainable reductions in all forms of tobacco use and associated morbidity and mortality (2,3).

These findings demonstrate a considerable gap between state investments in tobacco prevention and control and CDC’s Best Practices recommendations. Although all states derive revenues from cigarette excise taxes, few states have a statutory requirement requiring that a portion of these revenues be dedicated to tobacco prevention and control (10). Instead, most cigarette tax revenues are used for general purposes. Additionally, although in recent years state cigarette excise taxes have nationally increased, these tax increases largely have come in response to shortfalls in state budgets, rather than as initiatives to increase tobacco control spending (1,2). Many state programs have experienced and are facing substantial state government cuts to tobacco control funding, resulting in the near-elimination of tobacco control programs in those states (2). In 2014, despite combined revenue of more than $25 billion from settlement payments and tobacco taxes for all states, states have appropriated only $481.2 million (1.9%)†† to comprehensive tobacco control programs, an amount <15% of the CDC-recommended level of funding for all states combined (2). Only two states, Alaska and North Dakota, currently fund tobacco control programs at CDC-recommended levels.§§ Implementing comprehensive tobacco control programs at CDC-recommended levels could have a substantial impact: millions fewer persons in the United States would smoke and hundreds of thousands of premature tobacco-related deaths could be prevented; long-term investments could have even greater effects (2,3).

**Summary**

What is already known on this topic?

Evidence-based, statewide tobacco control programs that are comprehensive, sustained, and accountable reduce smoking rates and tobacco-related diseases and deaths. States that made larger investments in tobacco prevention and control saw larger declines in cigarettes sales than the United States as a whole. The prevalence of smoking has declined faster as spending for tobacco control programs has increased.

What is added by this report?

In fiscal year 2011, for tobacco prevention and control activities, all 50 states and the District of Columbia combined spent $658 million ($2.11 per capita) in the following categories: 41.4% on state and community interventions ($272 million [$0.87 per capita]); 18.8% on health communication interventions ($124 million [$0.40 per capita]); 20.4% on cessation interventions ($134 million [$0.43 per capita]); 9.3% on surveillance and evaluation ($61 million [$0.20 per capita]); and 10.1% on surveillance and evaluation ($67 million [$0.21 per capita]). The total spent was 17.8% of CDC’s recommended amount.

What are the implications for public health practice?

State investments in tobacco prevention and control programs in fiscal year 2011 were considerably less than levels recommended in CDC’s Best Practices. Full implementation of comprehensive tobacco control policies and evidence-based interventions at CDC-recommended funding levels could result in a substantial reduction in tobacco-related morbidity and mortality and billions of dollars in savings from averted medical costs and lost productivity in the United States.

The findings in this report are subject to at least three limitations. First, some expenditure data might not have been captured because it was spent by agencies or organizations that were not tracked, which could result in underestimation. For example, direct service expenditures on cessation by private insurers were not captured, neither were the direct expenditures on cessation made by state Medicaid in most states. However, aggregated state tobacco control expenditures were comparable with state tobacco control funding data reported elsewhere (10). Second, expenditure data were self-reported. As a result, variations might exist with regard to expenditure classifications across states. Finally, private organizations or foundations using private funds to conduct tobacco prevention and control activities were not included in the reported expenditures, which would lead to underestimation.

Each day in the United States, the tobacco industry spent nearly $24 million to advertise and promote cigarettes and smokeless tobacco (6,7). During the same period, more than 3,200 youth younger than 18 years of age smoked their first cigarette and another 2,100 youth and young adults who are occasional smokers progressed to become daily smokers (3). If current rates continue, 5.6 million Americans younger than 18 years of age who are alive today are projected to die prematurely from smoking-related disease (3). However, the tobacco-use epidemic can be markedly reduced by implementing interventions that are known to work. Full implementation of comprehensive tobacco control policies and evidence-based interventions at CDC-recommended funding levels could result in a substantial reduction in tobacco-related morbidity and mortality and billions of dollars in savings from averted medical costs and lost productivity in the United States (2,3).

## Figures and Tables

**TABLE 1:** National and state tobacco prevention and control expenditures, by program component, fiscal year 2011

	Program component (million $)					
	
State	Total spending	State/Community	Health communication	Cessation	Surveillance/Evaluation	Administration/Management
United States	$658.15	$272.38	$123.53	$134.09	$61.35	66.79
Alabama	9.01	5.69	0.56	1.83	0.24	0.68
Alaska	10.66	4.44	1.82	2.56	0.97	0.88
Arizona	19.15	7.85	3.61	4.42	0.45	2.83
Arkansas	13.38	5.97	1.37	3.51	1.02	1.51
California	94.66	41.09	15.01	7.27	21.17	10.12
Colorado	29.15	17.68	0.92	2.58	4.35	3.62
Connecticut	1.69	0.65	0.40	0.49	0.09	0.05
Delaware	9.30	4.30	1.00	1.00	1.40	1.60
DC	2.47	0.92	0.66	0.36	0.16	0.37
Florida	61.29	16.86	20.53	15.77	5.36	2.78
Georgia	3.46	1.02	0.44	1.13	0.33	0.54
Hawaii	8.05	3.25	1.73	1.36	0.63	1.08
Idaho	3.09	0.51	0.91	0.95	0.24	0.48
Illinois	15.87	8.76	1.12	3.82	0.77	1.41
Indiana	9.35	5.99	0.90	1.00	0.56	0.90
Iowa	8.03	3.94	1.75	1.58	0.20	0.55
Kansas	2.64	1.68	0.09	0.19	0.07	0.61
Kentucky	4.33	2.75	0.16	0.67	0.16	0.60
Louisiana	11.15	3.80	3.44	1.87	0.65	1.39
Maine	7.60	1.40	1.38	2.85	1.20	0.78
Maryland	6.02	2.43	0.00	2.41	0.45	0.73
Massachusetts	6.48	3.22	0.63	1.83	0.65	0.16
Michigan	5.93	2.87	0.33	1.33	0.21	1.20
Minnesota	19.63	6.42	4.69	2.98	2.31	3.22
Mississippi	11.70	5.56	2.00	1.73	0.96	1.45
Missouri	10.03	3.24	1.79	2.38	1.11	1.51
Montana	8.24	4.91	1.27	1.17	0.04	0.85
Nebraska	4.11	2.33	0.59	0.29	0.17	0.73
Nevada	5.84	1.96	2.00	0.79	0.16	0.93
New Hampshire	1.68	0.31	0.10	0.85	0.15	0.28
New Jersey	3.59	1.50	0.64	0.63	0.00	0.83
New Mexico	7.83	2.26	1.92	2.07	0.37	1.22
New York	57.67	20.22	17.77	16.73	0.72	2.23
North Carolina	20.40	10.54	4.84	2.13	1.93	0.97
North Dakota	7.68	3.45	0.87	2.61	0.37	0.38
Ohio	3.98	0.56	0.72	1.90	0.23	0.57
Oklahoma	24.72	6.77	5.13	7.28	2.04	3.50
Oregon	9.34	5.46	2.07	0.85	0.46	0.51
Pennsylvania	22.06	9.15	2.92	6.81	1.26	1.93
Rhode Island	3.84	1.01	0.64	0.71	0.34	1.14
South Carolina	4.04	1.84	0.20	1.36	0.07	0.57
South Dakota	4.88	1.20	0.63	2.43	0.22	0.40
Tennessee	2.12	0.87	0.35	0.50	0.08	0.31
Texas	18.67	8.82	3.63	3.48	0.96	1.79
Utah	8.39	2.93	1.59	1.80	0.91	1.16
Vermont	4.52	2.06	1.03	0.99	0.33	0.10
Virginia	12.06	2.14	4.15	1.79	1.88	2.10
Washington	17.48	9.95	0.79	4.16	1.24	1.34
West Virginia	7.20	2.55	1.30	2.20	0.57	0.58
Wisconsin	7.67	4.85	0.42	1.47	0.43	0.51
Wyoming	6.03	2.50	0.75	1.22	0.73	0.84

**Abbreviation:** DC = District of Columbia.

**TABLE 2:** Per capita national and state tobacco prevention and control expenditures, by program component, fiscal year 2011

	Program component ($)					
	
State	Total spending	State/Community	Health communication	Cessation	Surveillance/Evaluation	Administration/Management
United States	2.11	0.87	0.40	0.43	0.20	0.21
Alabama	1.88	1.18	0.12	0.38	0.05	0.14
Alaska	14.74	6.14	2.52	3.54	1.34	1.21
Arizona	2.95	1.21	0.56	0.68	0.07	0.44
Arkansas	4.55	2.03	0.47	1.19	0.35	0.52
California	2.51	1.09	0.40	0.19	0.56	0.27
Colorado	5.70	3.45	0.18	0.50	0.85	0.71
Connecticut	0.47	0.18	0.11	0.14	0.03	0.01
Delaware	10.25	4.74	1.10	1.10	1.54	1.76
DC	4.00	1.49	1.06	0.59	0.27	0.60
Florida	3.22	0.88	1.08	0.83	0.28	0.15
Georgia	0.35	0.10	0.04	0.12	0.03	0.06
Hawaii	5.85	2.36	1.26	0.99	0.46	0.79
Idaho	1.95	0.32	0.57	0.60	0.15	0.30
Illinois	1.23	0.68	0.09	0.30	0.06	0.11
Indiana	1.43	0.92	0.14	0.15	0.09	0.14
Iowa	2.62	1.29	0.57	0.52	0.07	0.18
Kansas	0.92	0.58	0.03	0.07	0.03	0.21
Kentucky	0.99	0.63	0.04	0.15	0.04	0.14
Louisiana	2.44	0.83	0.75	0.41	0.14	0.30
Maine	5.72	1.05	1.04	2.14	0.90	0.58
Maryland	1.03	0.42	0.00	0.41	0.08	0.13
Massachusetts	0.98	0.49	0.10	0.28	0.10	0.02
Michigan	0.60	0.29	0.03	0.13	0.02	0.12
Minnesota	3.67	1.20	0.88	0.56	0.43	0.60
Mississippi	3.93	1.87	0.67	0.58	0. 32	0.49
Missouri	1.67	0.54	0.30	0.40	0.18	0.25
Montana	8.26	4.92	1.27	1.17	0.04	0.86
Nebraska	2.23	1.26	0.32	0.16	0.09	0.40
Nevada	2.15	0.72	0.74	0.29	0.06	0.34
New Hampshire	1.28	0.23	0.08	0.64	0.11	0.21
New Jersey	0.41	0.17	0.07	0.07	0.00	0.09
New Mexico	3.76	1.08	0.92	0.99	0.18	0.59
New York	2.96	1.04	0.91	0.86	0.04	0.11
North Carolina	2.11	1.09	0.50	0.22	0.20	0.10
North Dakota	11.23	5.04	1.27	3.82	0.54	0.55
Ohio	0.34	0.05	0.06	0.16	0.02	0.05
Oklahoma	6.52	1.79	1.35	1.92	0.54	0.92
Oregon	2.41	1.41	0.53	0.22	0.12	0.13
Pennsylvania	1.73	0.72	0.23	0.53	0.10	0.15
Rhode Island	3.65	0.96	0.61	0.68	0.32	1.08
South Carolina	0.86	0.39	0.04	0.29	0.01	0.12
South Dakota	5.92	1.45	0.76	2.95	0.27	0.49
Tennessee	0.33	0.14	0.05	0.08	0.01	0.05
Texas	0.73	0.34	0.14	0.14	0.04	0.07
Utah	2.98	1.04	0.56	0.64	0.32	0.41
Vermont	7.21	3.29	1.64	1.58	0.53	0.16
Virginia	1.49	0.26	0.51	0.22	0.23	0.26
Washington	2.56	1.46	0.12	0.61	0.18	0.20
West Virginia	3.88	1.37	0.70	1.19	0.31	0.31
Wisconsin	1.34	0.85	0.07	0.26	0.07	0.09
Wyoming	10.62	4.41	1.31	2.15	1.29	1.47

**Abbreviation:** DC = District of Columbia.

**TABLE 3:** National and state tobacco control and prevention expenditures as a percentage of 2007 CDC-recommended levels, by program component, fiscal year 2011

	Program component (% of CDC-recommended levels)					
	
State	Total spending	State/Community	Health Communication	Cessation	Surveillance/Evaluation	Administration/Management
United States	17.8	18.6	17.5	12.8	19.1	41.5
Alabama	15.9	24.5	7.2	10.0	5.0	27.3
Alaska	99.6	83.7	130.0	98.4	107.3	175.0
Arizona	28.1	27.1	35.7	22.0	7.7	94.3
Arkansas	36.8	39.0	27.4	31.0	31.8	94.6
California	21.4	24.1	13.7	7.0	55.1	52.7
Colorado	53.6	76.2	10.7	16.7	92.6	150.8
Connecticut	3.8	3.7	4.4	4.4	2.4	2.6
Delaware	66.9	76.8	30.3	31.3	116.7	266.7
DC	23.6	19.1	28.6	18.2	18.2	73.8
Florida	29.1	21.5	56.7	23.0	29.3	30.2
Georgia	3.0	2.3	1.8	3.5	3.2	10.6
Hawaii	52.9	45.7	91.2	32.4	48.1	154.9
Idaho	18.3	6.5	38.0	21.7	15.7	68.1
Illinois	10.1	13.8	4.1	8.3	5.6	20.8
Indiana	11.9	19.0	7.8	3.9	8.1	26.5
Iowa	21.9	24.6	36.5	14.3	6.4	34.3
Kansas	8.2	11.4	2.5	2.0	2.6	43.6
Kentucky	7.6	11.9	2.3	3.4	3.1	23.8
Louisiana	20.8	16.7	50.6	11.1	13.9	60.3
Maine	41.1	17.9	43.2	55.8	75.1	96.9
Maryland	9.5	9.9	0.0	13.2	8.2	26.1
Massachusetts	7.2	10.2	2.5	8.5	8.3	4.1
Michigan	4.9	5.8	1.9	3.4	2.0	22.6
Minnesota	33.6	26.0	51.5	17.5	45.3	129.0
Mississippi	29.8	35.2	32.3	14.3	28.2	85.5
Missouri	13.7	11.2	15.4	10.3	17.3	47.2
Montana	59.3	77.9	50.7	35.5	3.5	142.3
Nebraska	19.1	25.0	16.8	4.9	9.2	81.1
Nevada	18.0	14.5	37.1	8.4	5.8	66.4
New Hampshire	8.8	4.4	2.0	18.8	8.8	34.8
New Jersey	3.0	3.6	1.9	2.2	0.0	16.0
New Mexico	33.5	20.7	73.7	30.0	18.4	122.1
New York	22.7	22.5	26.9	25.7	3.2	20.0
North Carolina	19.1	24.6	29.9	6.3	20.7	21.1
North Dakota	82.6	73.4	72.6	118.7	46.4	93.8
Ohio	2.7	1.0	3.1	4.3	1.8	9.0
Oklahoma	54.9	35.1	106.8	48.5	52.3	175.0
Oregon	21.7	30.7	29.5	6.7	12.5	26.7
Pennsylvania	14.2	16.4	9.1	14.4	9.3	28.4
Rhode Island	25.3	15.1	23.8	18.7	26.2	162.4
South Carolina	6.5	9.0	1.2	8.2	1.2	21.1
South Dakota	43.2	21.7	41.7	86.9	22.1	80.2
Tennessee	3.0	3.1	3.3	2.1	1.4	10.1
Texas	7.0	7.7	8.4	4.7	4.1	15.4
Utah	35.5	25.3	43.0	34.6	43.1	115.7
Vermont	43.4	44.8	44.8	47.2	37.0	20.0
Virginia	11.7	6.4	13.9	6.8	20.9	46.6
Washington	26.0	34.4	8.6	20.4	21.1	46.3
West Virginia	25.9	24.5	22.8	27.2	23.8	48.3
Wisconsin	11.9	17.6	5.3	7.3	7.6	18.0
Wyoming	67.0	56.9	49.7	64.2	91.4	208.8

**Abbreviation:** DC = District of Columbia.

## Footnotes

* Settlement revenue data (2011) were obtained from the National Association of Attorneys General. Net state cigarette excise tax revenues data (2011) were obtained from *The Tax Burden on Tobacco, 2011*. Revenues not included are excise taxes collected on smokeless tobacco products, local excise taxes, and state or local sales taxes.

† Additional information available at http://www.lungusa2.org/slati/.

§ Additional information available at http://tobacconomics.org/research/methodology-state-tobacco-control-and-prevention-expenditures-fy-2008-2011.

¶ State and community interventions comprised those that encompass changing local and statewide smoke-free air policies, reducing exposure to secondhand smoke, eliminating tobacco-related disparities, or implementing community and school programs aimed at reducing youth tobacco use. Health communication interventions comprised those that addressed youth and adult tobacco use behavior through television, radio, billboard, print, or web-based advertising; media advocacy; health promotion activities; efforts to reduce or replace tobacco industry sponsorship and promotions, or messages targeted to specific audiences. Cessation interventions comprised state quitlines or other cessation services. Surveillance and evaluation efforts and resources comprised surveys and research that monitor tobacco-related attitudes, behaviors, and health outcomes. They also include evaluation of the achievement and effectiveness of various tobacco control program interventions and goals. Administration and management resources comprised salary and fringe benefits for personnel that manage and operate state tobacco control programs.

** This report analyzed the program components and recommendation funding levels from Best Practices-2007 instead of the more recent Best Practices-2014 because the former report contained the published funding recommendations that applied to fiscal year 2011.

†† Additional information available at https://www.tobaccofreekids.org/what_we_do/state_local/prevention_cessation/.

§§ Additional information available at http://www.tobaccofreekids.org/microsites/statereport2015/.

## References

[1] CDC (2007). Best practices for comprehensive tobacco control programs—2007.

[2] CDC (2014). Best practices for comprehensive tobacco control programs—2014.

[3] US Department of Health and Human Services (2014). The health consequences of smoking—50 years of progress: a report of the Surgeon General.

[4] Farrelly MC, Pechacek TF, Thomas KY, Nelson D (2008). The impact of tobacco control programs on adult smoking. Am J Public Health.

[5] Tauras JA, Chaloupka FJ, Farrelly MC (2005). State tobacco control spending and youth smoking. Am J Public Health.

[6] Federal Trade Commission (2013). Federal Trade Commission smokeless tobacco report for 2011.

[7] Federal Trade Commission (2013). Federal Trade Commission cigarette report for 2011.

[8] Kann L, Kinchen S, Shanklin SL (2014). Youth risk behavior surveillance—United States, 2013. MMWR Surveill Summ.

[9] Jamal A, Agaku IT, O’Connor E, King BA, Kenemer JB, Neff L (2014). Current cigarette smoking among adults—United States, 2005–2013. MMWR Morb Mortal Wkly Rep.

[10] CDC (2012). State tobacco revenues compared with tobacco control appropriations—United States, 1998–2010. MMWR Morb Mortal Wkly Rep.

